# The biodistribution of triamcinolone acetonide injections in severe keloids: an exploratory three-dimensional fluorescent cryomicrotome study

**DOI:** 10.1007/s00403-024-03041-w

**Published:** 2024-06-08

**Authors:** Qi Yin, Vazula Z. Bekkers, Maud C. M. Roelofs, Johannes G. G. Dobbe, Judith de Vos, Paul R. Bloemen, Maurice C. G. Aalders, Susan Gibbs, Oren Lapid, Frank B. Niessen, Martijn B. A. van Doorn, Albert Wolkerstorfer

**Affiliations:** 1 Department of Dermatology, Amsterdam UMC location University of Amsterdam, Meibergdreef 9, Amsterdam, the Netherlands; 2 Department of Dermatology, Erasmus Medical Center, Dr. Molewaterplein 40, Rotterdam, The Netherlands; 3 Biomedical Engineering and Physics, Amsterdam UMC location University of Amsterdam, Meibergdreef 9, Amsterdam, the Netherlands; 4 Department of Molecular Cell Biology & Immunology, Amsterdam UMC location University of Amsterdam, Meibergdreef 9, Amsterdam, the Netherlands; 5 Amsterdam Movement Sciences, Amsterdam University Medical Center, Amsterdam, the Netherlands; 6 Department of Plastic, Reconstructive and Hand Surgery, Amsterdam UMC location Vrije Universiteit, De Boelelaan 1117, Amsterdam, The Netherlands

**Keywords:** Keloid, Triamcinolon, Cryomicrotome, Electronic pneumatic injection

## Abstract

Intralesional corticosteroid injections are a first-line treatment for keloids; yet clinical treatment results are highly variable and often suboptimal. Variation in triamcinolone acetonide (TAC) biodistribution may be an important reason for the variable effects of TAC treatment in keloids. In this exploratory study we investigated the biodistribution of TAC in keloids and normal skin using different drug delivery techniques. Fluorescent-labeled TAC suspension was administered into keloids and normal skin with a hypodermic needle and an electronic pneumatic jet injector. TAC biodistribution was represented by the fluorescent TAC volume and 3D biodistribution shape of TAC, using a 3D-Fluorescence-Imaging Cryomicrotome System. Twenty-one keloid and nine normal skin samples were analyzed. With needle injections, the mean fluorescent TAC volumes were 990 µl ± 479 in keloids and 872 µl ± 227 in normal skin. With the jet injector, the mean fluorescent TAC volumes were 401 µl ± 252 in keloids and 249 µl ± 67 in normal skin. 3D biodistribution shapes of TAC were highly variable in keloids and normal skin. In conclusion, TAC biodistribution in keloids is highly variable for both needle and jet injection. This may partly explain the variable treatment effects of intralesional TAC in keloids. Future research is needed to confirm this preliminary finding and to optimize drug delivery in keloids.

**Supplementary Information:**

The online version contains supplementary material available at 10.1007/s00403-024-03041-w.

## Introduction

Keloids are fibroproliferative scars caused by chronic inflammation in the reticular dermis. Keloids may cause pain, pruritus, movement restriction, and cosmetic concerns [1, 2]. They can be challenging to treat. Intralesional corticosteroid administration (ICA) by needle injection is traditionally considered a first-line treatment for keloids, with triamcinolone acetonide (TAC) being used most frequently [3, 4]. Nevertheless, clinical results of this treatment are highly variable and often suboptimal [5, 6].

Treatment efficacy can be influenced by various factors, such as the duration, size, anatomic location, genetic predisposition and treatment history of keloids [7]. Additionally, treatment efficacy is influenced by drug biodistribution, which may depend on characteristics of the tissue that is injected [8, 9]. Drug biodistribution may also depend on the drug delivery technique. Conventional needle injection using hypodermic needles has been used predominantly for ICA in the past few decades. Yet, a wide variation in this injection technique exists in current clinical practice [4]. Alternatively, different types of jet injectors can be used for ICA. Electronic pneumatic jet injectors, referred to hereinafter as ‘jet injectors’, are needle-free injectors that use pressured gasses (e.g. air or CO_2_) to create a high velocity jet stream of liquid drugs that penetrates the skin without using a needle [10]. The drug volume and gas pressure can be adjusted to match the dose and depth of drug administration to the specific clinical requirements.

To date, the biodistribution of TAC administered with different drug delivery techniques has not been investigated in different tissues. Variation in TAC biodistribution may be a major reason for the variable treatment effects of TAC in keloids reported in clinical trials and observed in clinical practice. The aim of this exploratory study is to assess the biodistribution of TAC in ex vivo keloids and normal skin with different drug delivery techniques.

## Materials and methods

Collection procedures were in compliance with article 7:467 BW of the Dutch law. Formal approval for this exploratory ex vivo study from the Medical Ethics Board Committee (METC) was not required, because the tissue samples only involved anonymously collected material that was discarded following routine elective surgery.

### Study design

In this exploratory study, the TAC biodistribution using different drug delivery techniques in ex vivo keloids and normal skin was investigated. TAC 40 mg/mL suspension (Kenacort, Bristol-Myers Squibb, New York City, New York, U.S.) was labeled with a fluorescent dye (Texas Red 10 µg/mL; 3000 MW, Invitrogen). TAC biodistribution was represented by the fluorescent TAC volume and 3D biodistribution shape of TAC, using a 3D-Fluorescence-Imaging Cryomicrotome System (3D-FICS).

The drug delivery techniques were (1a) needle injection in the superficial, mid, and deep layer of the keloid; (1b) perforation technique, i.e. making multiple cross-sectional passes with a thick needle prior to injection in the mid-layer of the keloid; and (2) jet injection using pressures of 4, 5 and 6 Bar. For jet injections, the residual TAC volume on the keloid and skin was determined by wiping off the fluid on the surface of the keloid and skin using a gauze and measuring the weight increase of the gauze. Then, this residual weight was converted to residual volume using a conversion rate of 1.0496 (1 mL TAC = 0.9527 g, based on own measurements). Papule formation after each jet injection was directly captured using a 3D-camera (LifeViz Micro 600D, Quantificare, Sophia Antipolis, France).

### Study samples

The selection of keloids was performed by two plastic surgeons (FN and OL) and one dermatologist (EP) experienced in keloid treatment. Keloids were included if a specimen of at least 1.5 × 1.5 cm could be harvested; regardless of the anatomic location, duration, etiology, and pretreatment. Keloids were excluded if (1) the differentiation between keloid and hypertrophic scar could not be made clinically, (2) the sample had been preserved in any preservative fluid, or (3) the patient had objected to the use of discarded material for scientific purposes.

The keloids were obtained from patients who underwent elective keloid excision and adjuvant radiotherapy (Department of Plastic Surgery Amsterdam UMC, Department of Dermatology Erasmus Medical Center, The Netherlands). Normal skin was obtained from patients who underwent abdominoplasty (Department of Plastic Surgery, Jan van Goyen Medical Center, The Netherlands). After removing excessive subcutaneous fat, the keloids and normal skin tissues were stored at -80 °C or at -20 °C, the latter for a maximum of 6 months.

### Experiments

Prior to the experiments, the normal skin and keloid samples were thawed to room temperature, fixed under mild tension, kept moist with wet gauzes, and marked with 1.5 × 1.5 cm zones. All experiments were conducted in triplicate, by a dermatologist experienced in ICA in keloids (AW).

For the experiments with ‘conventional’ needle, a 25-gauge needle and 1 mL syringe were used to administer 100 µL fluorescent-labeled TAC 40 mg/mL per sample. For the experiments with the jet injector, a needle-free jet injector (Enerjet 2.0, Perfaction, Rehovot, Israel) was used to administer 100 µL (device range: 50–130 µL) fluorescent-labeled TAC 40 mg/mL with pressures of 4, 5 and 6 bar (device range: 2–6 bar) per sample. All jet-assisted injections were administered perpendicularly in the center of the sample.

### Image acquisition

**Fig. 1:**
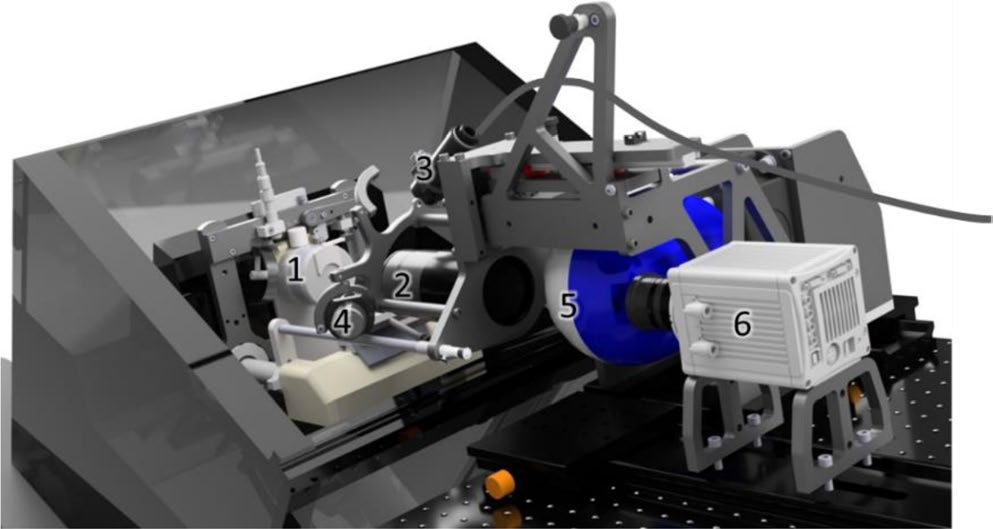
3D FICS. 1: Sample holder, 2: Lens (Olympus SZX16 stereo microscope system), 3: Tunable supercontinuum laser, 4: UV led illumination, 5: Tunable filter wheel, 6: Camera

The biodistribution of fluorescent-labeled TAC suspension was visualized using the 3D-FICS (Fig. 1). Firstly, all samples were embedded in a 3% carboxymethylcellulose with black ink to reduce background signaling. All samples were sectioned vertically into slices of 48 μm thickness. After each section, images were taken from the remaining bulk with a camera with an in-plane resolution of 13.66 × 13.66 μm. Prior to the experiments, wavelengths and exposure times were optimized using test samples. Eventually, a 595 nm excitation and 620 nm emission wavelength with an exposure time of 500 ms were used to visualize the fluorescent-labeled TAC suspension. A reflection image at 549 nm was used to reconstruct the tissue borders.

### Image analysis

**Fig. 2:**
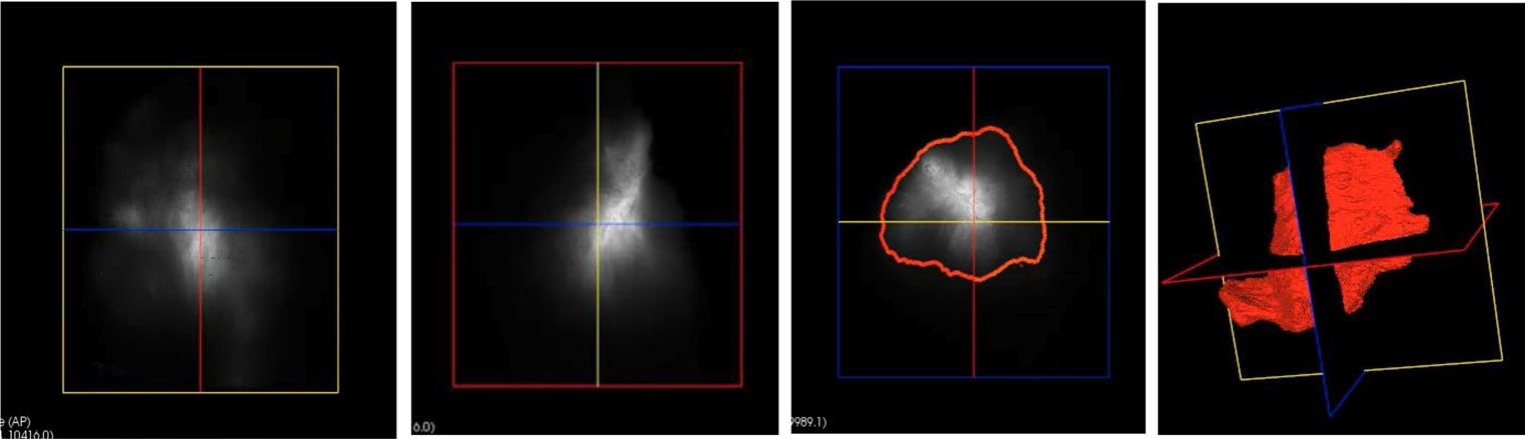
Fluorescent TAC biodistribution in keloid sample in axial, coronal and sagittal axis respectively; segmented fluorescent region representing the fluorescent TAC volume and the 3D biodistribution shape of TAC in a keloid sample

The 2D images were first cropped using LabVIEW (National Instruments, Austin, Texas, USA) and the resulting stack of images was resampled to a volume image with a resolution of (x, y, z) = 27.4 × 27.4 × 35.0 μm, which was sufficient to observe and quantify the fluorescent-labeled TAC. The 3D model of the fluorescent distribution (Fig. 2) was obtained by image segmentation using custom software (Dobbe, 2019). During segmentation, all voxels in the fluorescent region above a pragmatically chosen intensity threshold (2000) were included, while excluding voxels representing autofluorescent tissue as much as possible. The volume of the segmented fluorescent regions represents the fluorescent TAC volume. This is not equivalent to the actual injected TAC volume due to the point spread function of the imaging system and the chosen arbitrary intensity threshold. However, the measured fluorescent TAC volume enables comparison of the arbitrary fluorescent TAC volumes between samples.

## 3. Results

A total of 30 samples (21 keloid, 9 normal skin) were analysed for the biodistribution of TAC injected with a hypodermic needle and jet injector. The keloids were obtained from six patients who underwent elective keloid excision and adjuvant radiotherapy, and were located on the abdomen, chest, mandibula or shoulder. Normal skin was obtained from three patients who underwent abdominoplasty.

### Fluorescent TAC volume

**Fig. 3:**
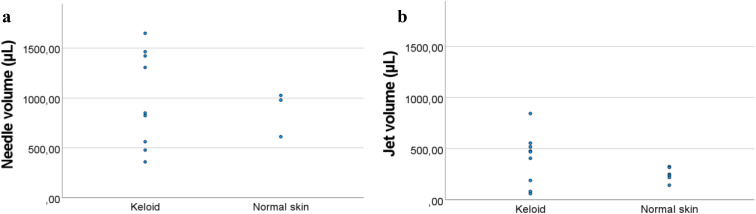
(**a**) Fluorescent TAC volumes in keloid and normal skin after needle injections. (**b**) Fluorescent TAC volumes in keloid and normal skin after jet injections

A large variation in fluorescent TAC volumes was observed in keloids (Fig. 3a and b). With the perforation technique, the mean fluorescent TAC volumes were similar (975 µl ± 284) compared to the ‘conventional’ needle injections (990 µl ± 479). Considerable operator injection force was needed for the needle injections and changing the needle position was sometimes necessary to inject the predefined volume of 100 µl.

With the jet injector, only a single attempt was made to inject the TAC. With the jet injector, the fluorescent TAC volume in keloids (401 µl ± 219) and normal skin (249 µl ± 59) seems to be smaller compared to needle injections (Tables 1 and 2).

**Table 1:** Mean fluorescent TAC volumes in mid-dermis of normal skin; and superficial, mid, and deep layers of keloid after needle injection

	Level of needle injection	Mean TAC volume (µL)
*Normal skin* (*n = 3*)	Middermal^1^	872 ± 227
*Keloid* (*n = 3*)	Superficial^1^	1069 ± 516
	Mid^1^	745 ± 159
	Deep^1^	1158 ± 697
	Perforation^2^	975 ± 284

^1^Standard technique, i.e. using one injection for TAC administration

^2^’Perforation technique’, i.e. making multiple cross-sectional passes with a thick needle prior to injection in the mid-layer of the keloid

Specified data is reported in Supplement 1

**Table 2:** Mean fluorescent TAC volumes and residual volumes in normal skin and keloid samples after jet injection. Specified data is reported in Supplement 2

	Jet pressure (Bar)	Mean TAC volume (µl)	Mean residual volume (%)
*Normal skin* (*n = 3*)	4	210 ± 59	28 ± 24
	6	287 ± 59	27 ± 8
*Keloid* (*n = 3*)	4	329 ± 447	59 ± 12
	5	384 ± 184	80 ± 12
	6	489 ± 27	89 ± 20

### 3D biodistribution shape

**Fig. 4:**
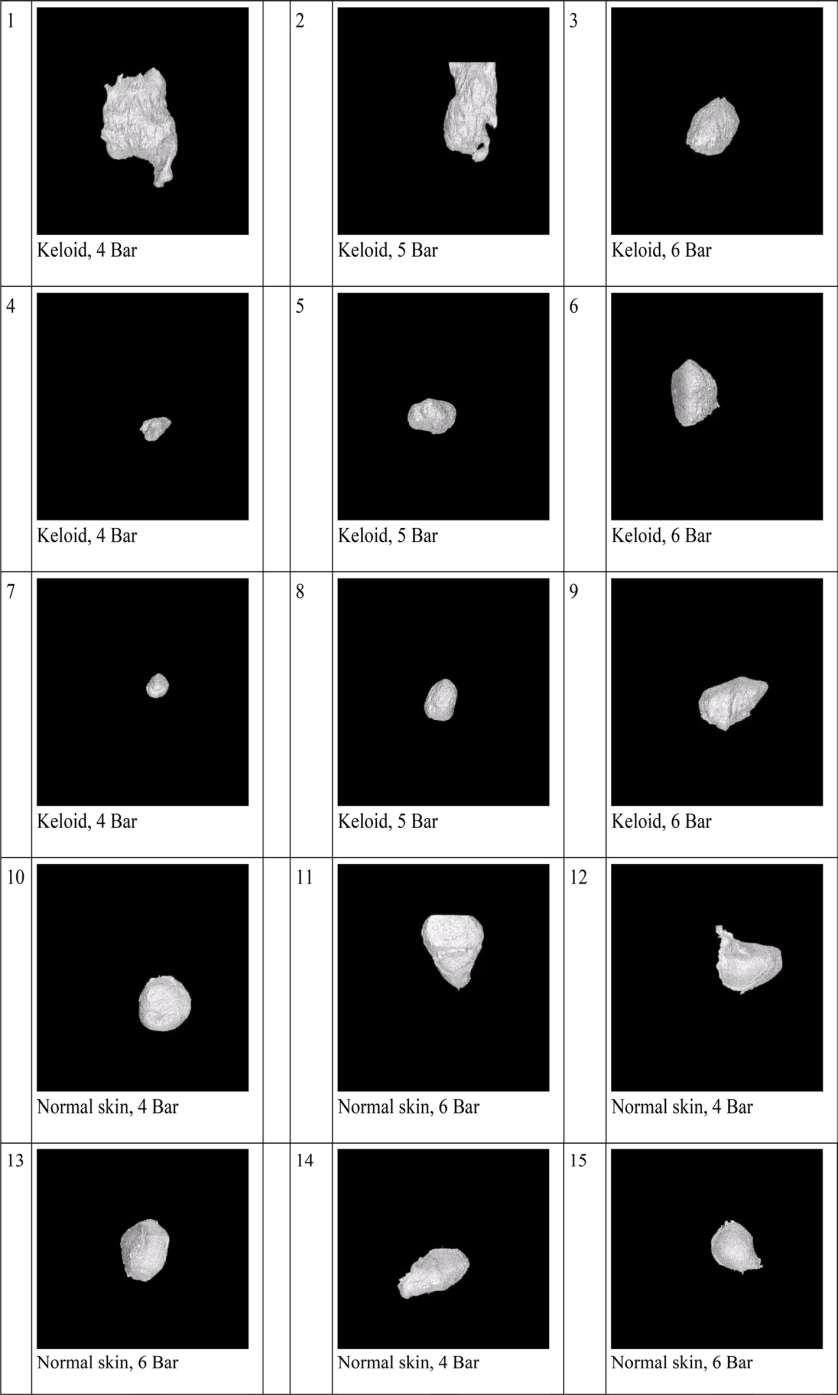
3D images of segmented fluorescent regions representing the TAC volume using a jet injector in keloid samples (1 to 9), and normal skin (10 to 15)

**Fig. 5:**
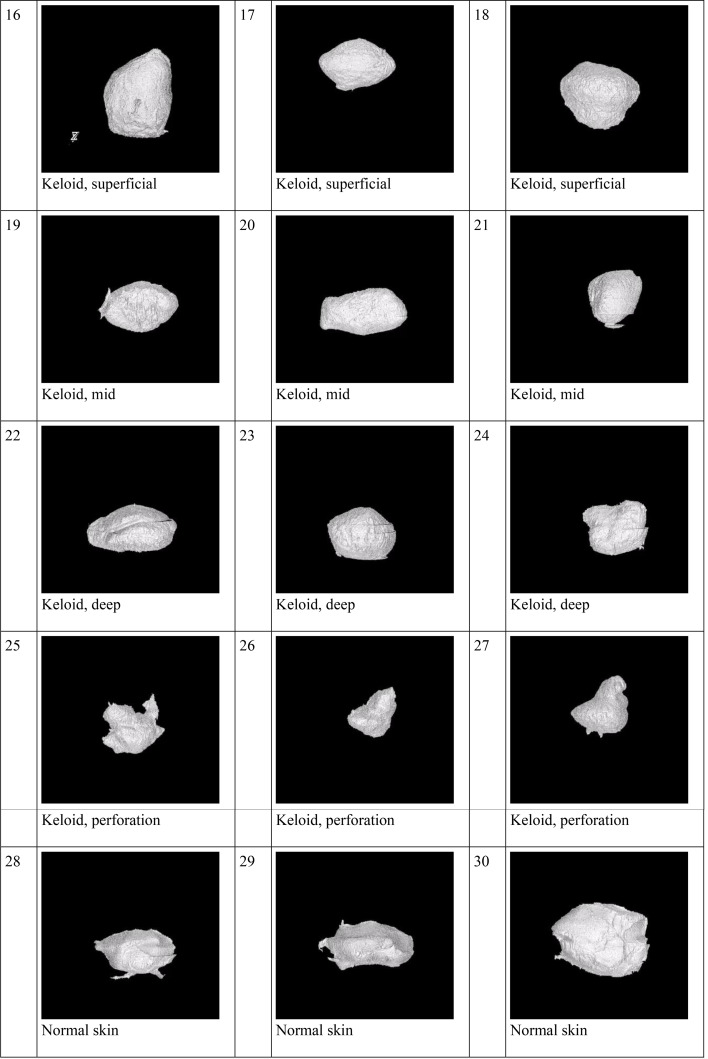
3D images of segmented fluorescent regions representing the TAC volume using needles in keloid samples (16 to 27), and normal skin (28 to 30)

The 3D biodistribution shape of TAC in keloids and normal skin using needle and jet injectors was highly variable (Figs. 4 and 5).

### Clinical endpoints jet injections

The residual TAC volume on the skin surface after jet injections was high, especially in keloids (Table 2). Using higher pressure of 6 Bar seems to result in larger fluorescent TAC volumes in keloids, compared to lower pressures (Table 2). Contradictory, higher pressure also resulted in larger residual volumes in our experiments. Papule formation was observed in 66% (6/9) of the keloid samples, while this was observed in all (6/6) normal skin samples. No clear relation was observed between papule formation in keloids and mean fluorescent TAC volume.

## Discussion

To date, studies about the treatment of keloids have focused on the various drugs for intralesional administration while the challenges and limitations of optimal biodistribution in keloids have been neglected. In this exploratory study, the 3D biodistribution of TAC using different drug delivery techniques in ex vivo keloids and normal skin was assessed using the 3D-FICS.

Large heterogeneity in TAC volumes was observed in keloids. This may be the result of the large variation in mechanical properties such as rigidity and viscoelasticity among different keloids and even within the same keloid. This variation in mechanical properties may depend on the anatomic location, prior treatment and genetic predisposition (1, 3, 22). Moreover, with jet injectors the fluorescent TAC volume seems to be smaller compared to needle injections. This is in line with the large residual TAC volume remaining on the skin surface of keloids (71.9 µl ± 14.3) after jet injection. Pressures generated by the jet injector may not be sufficient for penetrating the recalcitrant solid keloids in this ex vivo setting. Notably, the considerable operator injection force as applied in the experiments with needle injection could only be possible in clinical practice if prior local anesthesia has been applied, as it could be very painful otherwise. And for jet injections, repeating the injection would preferably be performed in clinical practice if a high amount of residual fluid is observed directly after jet injection. For needle injections, blanching is an endpoint of infiltration in clinical setting. However, this could not be used as a reference in these experiments using ex vivo samples without blood perfusion.

Compared to lower pressures, a higher pressure of 6 Bar seems to result in larger fluorescent TAC volumes in keloids. It should be emphasized that fluorescent TAC volumes are not equivalent to actual delivered TAC volumes. We assume that even though 6 Bar may result in larger fluorescent TAC distribution compared to lower pressures, the actual delivered TAC dose was lower, as reflected by the larger residual volumes. The reason for the latter is unclear; and the effect of different pressure levels on the biodistribution in keloids needs further investigation.

Considering the 3D biodistribution shape of TAC, we noticed substantial heterogeneity. These variable patterns of 3D TAC shapes contribute to the observation of the large heterogeneity in TAC biodistribution and ultimately clinical response.

There are several strengths to this study. To the best of our knowledge, there are no similar studies assessing the 3D biodistribution of TAC in keloids. Moreover, several drug delivery methods were studied, including needle injection, perforation technique, and jet injection. Furthermore, an innovative 3D imaging technique was used. Various imaging techniques may be used for assessing layers of the skin, including confocal microscopy, optical coherence tomography and high frequency ultrasound. In contrast to these imaging techniques, the custom-built 3D-FICS can be used for high resolution segmentation of large 3D volumes [11]. This novel imaging technique has previously been used in other medical specialties including cardiology to visualize the perfusion distribution within the heart and in neurology for imaging fluid distribution in brain structures [12, 13].

However, there are also several limitations to this study. Firstly, the sample size was limited, because keloids are excised infrequently. Secondly, differentiation in keloid characteristics such as anatomical location, tissue density and prior treatment was not performed due to the small sample size. Moreover, the included keloids were selected for excision and adjuvant brachytherapy, and differ from the usually smaller, thinner and less rigid keloids in clinical practice. Additionally, we visualized the fluorescent marker that was labeled to the TAC suspension, being a proxy for the TAC suspension. Although the fluorescent TAC volumes are not equivalent to the actual injected TAC volumes, they enabled comparison of the fluorescent biodistribution between samples. Furthermore, the observed fluorescent TAC volume and 3D biodistribution shape could be affected by the optical properties of tissue types, which may be different for normal and keloid tissue. Finally, inherent to the exploratory design of the study, *in-vivo* conditions such as blood flow, skin turgor, and hydration of the skin could not be taken into consideration.

Despite the above-mentioned limitations, the preliminary findings of this exploratory study are important for the clinical practice. Improving knowledge of drug biodistribution in keloids is not only important to enhance efficacy and safety for TAC, but also for other intralesionally administered drugs such as 5-fluorouracil and bleomycin. This exploratory study provides a framework for future studies on drug distribution. Future research may focus on improving biodistribution in keloids, either by changing the device for injection, injection technique of the physician (e.g. level of injection, perforation technique) or mechanical tissue properties (e.g. by hyperthermia, cryotherapy, radiotherapy and lasers). Interestingly, previous research in tumors demonstrated better biodistribution of certain tumor drugs when applied with a multiside hole needle (22 holes at the side of the needle) instead of the conventional ‘end hole needle’ [8]. Other future research challenges to better understand drug biodistribution are the measurement of absolute TAC volumes, concentrations and dimensions. A quantitative technique such as ELISA could be used to measure the drug concentrations in different skin levels.

In conclusion, our experiments indicate that TAC biodistribution in keloids is highly variable for both needle and jet injection. This may partly explain the variable treatment effects of intralesional TAC in keloids. Moreover, with jet injectors the fluorescent TAC volume seems to be smaller compared to needle injection in keloids. However, more experiments are needed to confirm the findings of this exploratory study.

## Electronic supplementary material

Below is the link to the electronic supplementary material.

Supplementary Material 1

## Data Availability

The datasets generated during and/or analysed during the current study are available from the corresponding author on reasonable request.
