# Supplementary material for: The biodistribution of triamcinolone acetonide injections in severe keloids: an exploratory three-dimensional fluorescent cryomicrotome study
Source: Arch Dermatol Res. 2024 Jun 8;316(7):368. doi: 10.1007/s00403-024-03041-w (PMC11162396; doi:10.1007/s00403-024-03041-w)
Supplement: Supplementary file 1 — Supplementary Material 1 [file 403_2024_3041_MOESM1_ESM.docx]

**Supplementary files**

| Tissue (location) | Location of injection | TAC volume (µL) |
| --- | --- | --- |
| Keloid (Abdomen) | S | 477.6 |
| Keloid (Back) | S | 1422.1 |
| Keloid (Chest) | S | 1306.3 |
| Keloid (Abdomen) | M | 562.1 |
| Keloid (Back) | M | 823.6 |
| Keloid (Chest) | M | 849.4 |
| Keloid (Chest) | M^1^ | 648.7 |
| Keloid (Chest) | M^1^ | 1109.9 |
| Keloid (Chest) | M^1^ | 1166.8 |
| Keloid (Abdomen) | D | 359.8 |
| Keloid (Back) | D | 1463.6 |
| Keloid (Chest) | D | 1649.1 |
| Normal skin | M | 612.0 |
| Normal skin | M | 978.6 |
| Normal skin | M | 1026.0 |

**Supplement 1**. Needle injections: location of injection (S: superficial, M: mid, D: deep) and TAC volume (µL).

^1^’Perforation technique’, i.e. making multiple cross-sectional passes prior to injection in the mid-layer of the keloid through one of the passes.

| Tissue (location) | Pressure (Bar) | TAC volume (µL) | Residual volume (%) |
| --- | --- | --- | --- |
| Keloid (Chest) | 4 | 844.6 | 55.5 |
| Keloid (Mandibula 1) | 4 | 81.8 | 72.3 |
| Keloid (Mandibula 2) | 4 | 60.8 | 48.9 |
| Keloid (Chest) | 5 | 555.1 | 69.3 |
| Keloid (Mandibula 1) | 5 | 406.7 | 93.8 |
| Keloid (Mandibula 2) | 5 | 189.9 | 78.2 |
| Keloid (Chest) | 6 | 477.2 | 112.3 |
| Keloid (Mandibula 1) | 6 | 519.2 | 74.9 |
| Keloid (Mandibula 2) | 6 | 470.3 | 82.1 |
| Normal skin | 4 | 142.9 | 55.4 |
| Normal skin | 4 | 250.3 | 11.8 |
| Normal skin | 4 | 238.0 | 17.5 |
| Normal skin | 6 | 324.7 | 18.5 |
| Normal skin | 6 | 317.1 | 27.9 |
| Normal skin | 6 | 219.1 | 34.6 |

**Supplement 2.** Jet injections: used pressures (Bar), TAC volumes (µL) and ‘residual volume’ (%).
